# Nona-Arginine Mediated Anti-E6 ShRNA Delivery Suppresses the Growth of Hela Cells *in vitro*

**DOI:** 10.61186/ibj.3963

**Published:** 2023-08-15

**Authors:** Razieh Taghizadeh Pirposhteh, Ehsan Arefian, Arash Arashkia, Nasir Mohajel

**Affiliations:** 1 Department of Molecular Virology, Pasteur Institute of Iran, Tehran 1316943551, Iran;; 2 Department of Microbiology, School of Biology, College of Science, University of Tehran 1417614411, Iran

**Keywords:** Cell-penetrating peptides, E6 oncogene, Human papillomavirus 18, RNA interference

## Abstract

**Background:**

The E6 oncoprotein of HPV plays a crucial role in promoting cell proliferation and inhibiting apoptosis, leading to tumor growth. Non-viral vectors such as nona-arginine (R9) peptides have shown to be potential as carriers for therapeutic molecules. This study aimed to investigate the efficacy of nona-arginine in delivering E6 shRNA and suppressing the E6 gene of HeLa cells in vitro.

**Methods:**

HeLa cells carrying E6 gene were treated with a complex of nona-arginine and E6 shRNA. The complex was evaluated using gel retardation assay and FESEM microscopy. The optimal N/P ratio for R9 peptide to transfect HeLa cells with luciferase gene was determined. Relative real-time PCR was used to evaluate the efficiency of mRNA suppression efficiency for E6 shRNA, while the effect of E6 shRNA on cell viability was measured using an MTT assay.

**Results:**

The results indicated that R9 efficiently binds to shRNA and effectively transfects E6 shRNA complexes at N/P ratios greater than 30. Transfection with R9 and PEI complexes resulted in a significant toxicity compared to the scrambled plasmid, indicating selective toxicity for HeLa cells. Real-time PCR confirmed the reduction of E6 mRNA expression levels in the cells transfected with anti-E6 shRNA.

**Conclusion:**

The study suggests that R9 is a promising non-viral gene carrier for transfecting E6 shRNA in vitro, with significant transfection efficiency and minimal toxicity.

## INTRODUCTION

Cervical cancer with 604,127 new cases and 341,831 deaths in 2020 is considered the fourth most common cancer in the female population worldwide. Furthermore, an estimated 14,000 women in the United States are diagnosed with this disease each year^[^^1^^]^. For the first time in 1983, Dürst et al.^[^^2^^]^ demonstrated a link between HPV and various cancers, especially cervical cancer. Epidemiological studies have shown that HPV persistent infection causes more than 95% of all cervical cancers. Among the different HPV genotypes, HPV16 and HPV18, as two high-risk genotypes, are found in 70-75% of cervical cancer biopsies^[^^3^^,^^4^^]^. 

The genome of the HPV encodes two types of proteins; delayed and early proteins. Among the early proteins, E6 and E7 oncoproteins have different oncogenic activities and are more necessary for malignant conversion^[^^5^^]^. The tumor induction mechanism of E6 and E7 is due to the high affinity of these oncoproteins for binding to tumor suppressors p53 and pRB^[^^6^^]^. The E6 protein forms a trimeric complex including E6 (the tumor suppressor protein p53) and E6-AP (the cellular ubiquitination enzyme). This complex stimulates the degradation of p53 and then disrupts the cell cycle, promotes cell proliferation and leads to the growth of tumor cells. In particular, selective suppression of E6 expression is desirable because E7-induced unregulated cell proliferation is expected to induce apoptosis without coincident suppression of p53 with E6^[^^7^^]^.

Natural RNA interference, a process for selective suppression of targeted genes with high specificity, is considered a potential tool for personalized cancer treatment^[^^8^^]^. siRNA and shRNA are among the molecules applied in the natural RNA interference approach. Generally, lower concentrations of shRNA (less than 5 copies), compared to siRNA (in the nM range), is required to knock down the gene expression, which in turn reduces off-target effects^[^^9^^,^^10^^]^. However, the same as other nucleic acid-based therapies, a suitable carrier is needed to protect and deliver shRNA to the target HPV-infected cells. Viral and non-viral nucleic acid delivery systems have been examined for the delivery of shRNAs. Despite the dominance of viral vectors in clinical trials, non-viral vectors are attractive due to their non-immunogenicity, low cost, reproducibility, and higher safety compared to viral vectors^[^^11^^]^. However, they suffer from low efficiency and intrinsic toxicity of their building blocks, e.g. positively charged lipids and monomers of the synthesized polymers. CPPs offer greater stability and biocompatibility than other non-viral vectors. Despite their low molecular weight, these peptides can provide different functions such as forming complexes with nucleic acids, cell penetration, and endosomal disruption, all of which are crucial for gene delivery^[^^12^^-^^14^^,^^15^^]^. Studies have demonstrated that nona-arginine (R9) can non-covalently bind to siRNA and deliver it into mammalian cells^[^^16^^,^^17^^]^. E6 inhibition by siRNA delivery to HPV-infected cells has been reported^[^^18^^,^^19^^]^. Despite their potential, peptide delivery systems have to be tested for the ability to deliver siRNA or shRNA to these cells in order to target E6 oncogene.

Herein, we presented evidence that the R9 peptide can deliver E6 shRNA (plasmid carrying anti-E6 shRNA) into HeLa cells. Our results demonstrated that R9 formed stable complexes with pDNA, which exhibited appropriate size and surface charge. Moreover, R9 was able to transfer reporter gene or shRNA-coding sequence to HeLa cells with minimal non-specific toxicity and suppress E6 gene expression in these cells, which resulted in significant targeted toxicity.

## MATERIALS AND METHODS

**Peptide** **synthesis**

R9 peptide (Mw: 1.42 kDa) was costume synthesized by GenScript Company (USA). The purity of the peptide lyophilized powder was 96%, which was utilized without further processing.

**shRNA design**

BlockIT software (Invitrogen, USA) was employed to design anti-E6 shRNA using E6 reference sequence as input. The resulting sequences were evaluated based on their score and delta G values, in which the one with the best score and lowest delta G was chosen. The RNAfold web server (http://rna.tbi.univie.ac.at/cgi-bin/RNA WebSuite/RNAfold.cgi) was then used to analyze the selected sequences in terms of their location on the target mRNA stem or loop region. Ultimately, a shRNA with a greater number of nucleotides in the loop region was selected. The final shRNA sequence was as follows: 5'-GCGCGCTTTGAGGATCCAACACGAATGTTGG ATCCTCAAAGCGCGC-3'. The E6 shRNA sequence, downstream of the U6 promoter sequence and upstream of U6 terminator, were synthesized and cloned into the pUC57 plasmid (obtained from Bio Basic, Canada), hereinafter referred to 'E6 shRNA'. Scrambled shRNA sequence under the control of the U6 promotor was synthesized in the same plasmid. 

**Reporter gene**

A modified pgL4.17 vector with cytomegalovirus promoter and luciferase reporter gene was used throughout the physicochemical and biological experiments.

**Nanoparticle formation**

The R9 peptide was dissolved in pharmaceutical water for injection in order to achieve a concentration of 0.32 µg/µL as a peptide stock solution. The pH of the peptide stock solution was adjusted to a range of 6 to 7. The peptide-pDNA complexes were formed by the addition of 15 µL of peptide solutions, prepared by the dilution of the stock solution with water to achieve the specific amine-to-phosphate ratio, to 10 µL of pDNA solution (0.1 µg/µL) in Tris-EDTA buffer (pH 8.5). The mixture was pipetted repeatedly for 20-25 seconds and incubated at 4 °C for 25 minutes prior to experiments. 

**Gel retardation assay**

The gel retardation assay was used to assess the formation of stable complexes between the peptide and nucleic acid. Peptide-pDNA complexes were prepared in N/P ratios of 5 to 30, and gel retardation experiments were carried out by loading nano-complexes and naked pDNA (as the control) to the wells of 1% agarose gel and electroporation at 100 V in Tris-acetate-EDTA buffer for 30 minutes.

**Field emission scanning electron microscopy**

Droplets of peptide-pDNA complexes (5 µL of peptide/pDNA complex mixture diluted with ratio of 1 to 100 and N/P ratios of 15, 30, and 45) were deposited onto freshly cleaved mica substrates and left to rest close to open flame for 30 minutes and dried in a closed box overnight. Samples were cast onto copper grids coated with carbon film, and FESEM microscopy was performed using a FESEM instrument (ZEISS Sigma VP model, Germany) in the resolution of 1.3 nm at 1 kV. On average, the size of 40-50 particles per image was measured.

**Transfection**

Reporter gene luciferase assay was performed to evaluate the transfection efficiency of R9 peptide. About 15,000 HeLa cells were seeded in a 96-well plate, and after 17 hours, the cells were transfected with R9-pDNA nanocomplexes at a constant pDNA concentration of 0.1 µg/µL and various concentrations of R9 to achieve N/P ratios ranging from 20 to 60- in each well of 96-well plates. The cells were then incubated for 48 hours before being subjected to luciferase assay using the luciferase assay system (E1500, Promega, USA) and the emitted light was measured using Cytation 3 Cell Imaging Multi-Mode Reader (BioTek, USA). Similarly, complexes of pDNA with branched 25 kDa polyethyleneimine (Sigma-Aldrich, US) were prepared at N/P of 10 as control. The emitted light units were normalized using total protein content of the transfected cells measured by bicinchoninic acid assay (Thermo Fisher Scientific, USA). To investigate the inhibition of the E6 gene and apoptosis induction by transfecting the E6 shRNA plasmid into the HeLa cell line, we selected four experimental groups. The groups included (i) E6 shRNA-R9 nanocomplexes at N/P of 30, (ii) E6 shRNA-PEI nanocomplexes at N/P of 10, (iii) scrambled shRNA-R9 nano-complexes at the same N/P ratio, and (iv) scrambled shRNA-PEI nanocomplexes. HeLa cells were cultured in DMEM containing 10% FBS and 1% penicillin/streptomycin and seeded at a density of 250,000-500,000 cells per well in a 24-well plate. After incubating for 18 hours, the medium was removed, and the cells were washed with PBS before adding 400 μL of serum-free medium. Then 100 μL of nano-complex mixture was added to each well; 60 µL of R9 peptide solution was added slowly to 40 μL of E6 shRNA at the concentration of 0.1 µg/µL to form nanocomplexes with N/P of 30. The plate was incubated in a cell culture incubator at 37 °C for 4-5 hours. After incubation, 100 µL of DMEM with 60% FBS was added to each well to obtain 10% FBS concentration in the cell culture media. The cells were then incubated for approximately 48 hours. The medium was then removed, and the cells were washed with PBS. 

**Real-time PCR**

Real-time PCR was utilized to determine the mRNA suppression efficiency of the gene of interest after transfection with the E6 shRNA compared to the untreated cells. Real-time PCR primers were designed, and the sequences were as follow: forward primer: 5’-ATCAA CAACAACCAGTACGG-3’ and reverse primer: 5’-GACGATGGGATGGGAATAC-3’. Beta-actin was targeted as a housekeeping gene by primer sequence of 5’-CACCAACTGGGACGACATG-3’ and 5’-GGCGT ACAGGGATAGCACA-3’ as forward and reverse primers, respectively. Total RNA was extracted using Hybrid-R™ kit (Geneall^®^, Korea), from the cells transfected with the shRNA, and cDNA was synthesized (BioFact^TM^, Smart Science, Thailand) from each well, separately. Real-time PCR was performed with the prepared cDNA by SYBR green master mix (Ampliqon, Paris, France) using real-time PCR (Applied Biosystems, USA). Instrument settings were programmed according to master mix protocol and analyzed using the 2^-∆∆ct ^method, to evaluate the recreation of E6 gene expression relative to the expression level of control cells. 

**Cytotoxicity assay**

This study consisted of two types of toxicity evaluations. The first involved assessing the toxicity of R9 peptide with N/P ratios ranging from 20 to 60, and PEI with N/P ratio of 10. The second evaluation was focused on the targeted toxicity of E6 shRNA when transfected with PEI and R9. The tests were performed through transfection, followed by a cytotoxicity assessment using the MTT kit (Life Biolab, Germany). The cells were transfected and treated with the shRNA or control complexes in a 96-well plate. After 48 hours, the medium was replaced with 100 μL of a fresh medium. Then 10 μL of MTT solution (5 mg of MTT powder in 1 mL of PBS buffer) was added to each well and further incubated for 4 hours. The formazan crystals formed in viable cells were dissolved in dimethyl sulfoxide. The plate was covered with aluminum foil and mixed at 100 rpm for 15 minutes. Finally, the absorbance was measured at 570 nm using an Epoch microplate reader (BioTek, USA). The experiment was performed in triplicate. 

**Fig. 1:**
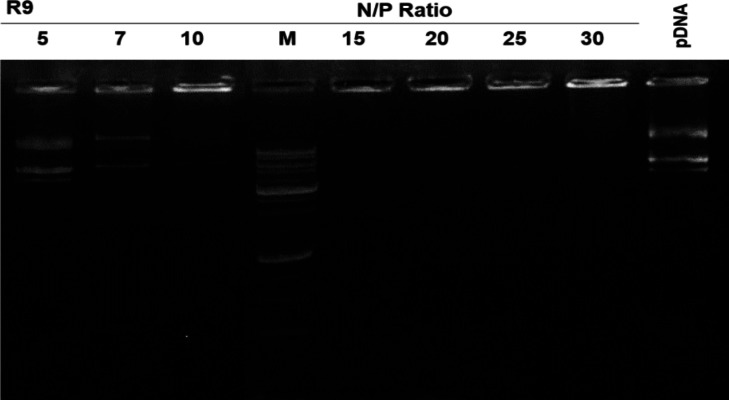
R9 peptide inhibiting pDNA movement in gel electrophoresis at N/P ratios equal to and higher than 15. Nanocomplexes were formed between peptide and pDNA at N/P ratios ranging from 5 to 30, and electrophoresis was conducted on a 1% w/v agarose gel. The marker was denoted as "M"

**Statistical analysis**

Statistical analysis using one-way ANOVA and Tukey’s post-hoc test was performed in GraphPad Prism version 8 for Windows (GraphPad Software, San Diego, California, USA) to examine the differences among the groups. p < 0.05 was considered as statistically significant.

## RESULTS

**R9 formed stable and nano-sized complexes with plasmid**

**Fig. 2:**
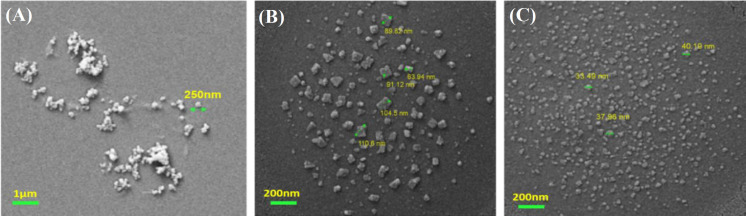
FESEM microscopy showing the morphology and size of R9 nanocomplexes with pDNA at N/P ratios of 15, 30, and 45. The size of the complexes was approximately (A) 250 nm at an N/P ratio of 15, (B) 80 nm at an N/P ratio of 30, and (C) less than 40 nm at an N/P ratio of 45, as determined by visual measurement of diameter for 45-50 particles. Particle sizes for a selected number of nano-complexes are displayed in the picture (green arrows)

The gel retardation assay showed that R9 effectively retarded pDNA in gel electrophoresis at N/P ratios equal to and greater than 15, indicating that R9 can efficiently bind to and compact pDNA. Further analysis using FESEM micrographs showed amorphous and irregular structures at an N/P ratio of 15. However, as the N/P ratio increased to 30 and 45, the complexes became more regular in their shape and smaller in size, decreasing from 250 nm at the N/P ratio of 15 to 40 nm at the N/P ratio of 45. This observation suggests that the size and morphology of the nano-complexes formed by R9 and pDNA were dependent on the N/P ratio (Figs. 1 and 2). 

**pDNA-R9 nanocomplexes transfected HeLa cells with minimal toxicity**

**Fig. 3:**
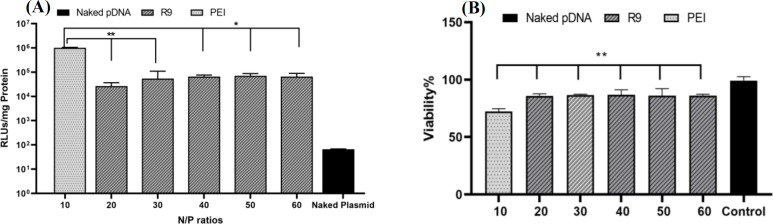
(A) Transfection efficiency of R9 nanocomplexes at N/P ratios ranging from 20 to 60. Luciferase assay was performed after transfection of HeLa cells with PEI and R9 nanocomplexes. R9 nanocomplexes exhibited unchanged transfection efficiencies at N/P ratio of 30 and more. Transfection efficiency of PEI at N/P ratio of 10 was higher than R9 at all N/P ratios. HeLa cells transfected with naked pGL4.17 were used as the control; (B) Toxicity of the transfected R9 peptide with N/P ratios ranging from 20 to 60, which was assessed to evaluate the toxicity of the reagent. R9 showed no significant changes in toxicity as the N/P ratio increased from 20 to 60. Results are reported as mean ± SD of three replicates. Results of the statistical analysis are shown by ^*^p < 0.05 and ^**^p < 0.01

Transfection efficiency of the R9 nanocomplexes was evaluated by measuring the relative light units emitted from luciferin normalized by total cell protein (Fig. 3A). Moreover, the toxicity of R9 at the same N/P ratios was compared to the toxicity of PEI at the N/P ratio of 10. The results of the experiments demonstrated that the R9 nanocomplexes were capable of successfully transfecting HeLa cells with pGL4.17 at an N/P ratio of 

30 or higher. There was no significant increase in transfection efficiency of R9 with further increases in the N/P ratio, while the transfection efficiency of PEI at the N/P ratio of 10 was significantly higher than R9 at all N/P ratios (p < 0.01 in comparison to N/P of 20 and 30 and p < 0.05 compared with N/P 40, 50, and 60). However, this peptide showed significantly lower toxicities at all N/P ratios on HeLa cells compared to PEI at the N/P ratio of 10 (p < 0.01; Fig.3B). Also, the cytotoxicity of R9 did not significantly change as the N/P ratio increased from 20 to 60.

**The E6 gene expression suppressed by shRNA-R9 nano-complexes**

**Fig. 4:**
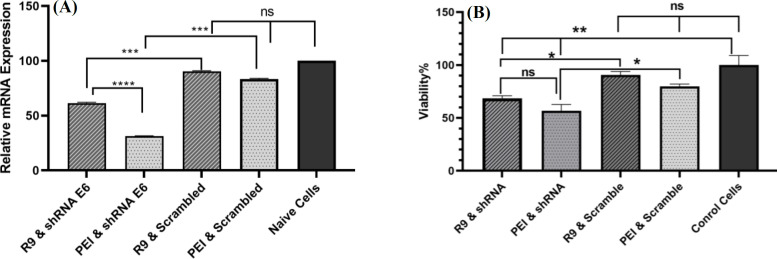
(A) Efficiency evaluation of E6 suppression via E6 shRNA transfection with PEI and R9 using real-time PCR, revealing a 52.39% and 28.76% reduction in E6 mRNA expression, respectively. Scrambled plasmid transfection using both PEI and R9 had no significant impact on E6 mRNA gene expression while E6 shRNA transfection with either PEI or R9, significantly reduced its expression. The suppression level was significantly higher when E6 shRNA was transfected with PEI (B)،Targeted toxicity of E6 shRNA was assessed by MTT test 48 h after transfection with PEI and R9. E6 shRNA showed a significant targeted toxicity on HeLa cells when transfected with R9, while PEI did not demonstrate significantly higher targeted toxicity compared to R9. Naive cells are HeLa cells without any treatments. Results are reported as mean ± SD of three replicates. Results of the statistical analysis are shown by * (p < 0.05), ** (p < 0.01), ***(p < 0.001), and **** (p< 0.0001)

Real-time PCR technique was used to assess the efficacy of shRNA in suppressing the expression of E6 mRNA. HeLa cell line transfection was performed in 24-well plates, followed by RNA extraction and cDNA synthesis from each well in three replicates (Fig. 4A). The results showed that the expression level of E6 mRNA in the cells transfected with shRNA by R9 peptide decreased by 1.4 folds (28.76%), while the expression decreased by 2.1 folds (52.39%) after being transfected with PEI. The scrambled shRNA did not result in statistically significant changes in expression (p > 0.05), indicating that the observed effects in reducing the expression of the E6 gene were specifically due to the effect of E6 shRNA. The higher level of E6 gene suppression in PEI-transfected cells can be attributed to its higher transfection efficiency compared to R9.

**E6 shRNA-R9 nano-complexes showed targeted toxicity on HeLa cells**

Targeted toxicity of E6 shRNA transfected with R9 and PEI on HeLa cells was assessed by MTT test (Fig. 4B). The E6 shRNA transfected with R9 and PEI complexes on HeLa cells demonstrated significantly higher toxicity than the scrambled shRNA (p < 0.05). Furthermore, this difference was not significant between transfected scrambled plasmid and normal cells (p > 0.05). E6 shRNA did not reveal a significantly higher targeted toxicity on HeLa cells when transfected with PEI compared to R9 (p > 0.05).

## DISCUSSION

In the present study, we investigated an R9-mediated shRNA delivery system. Results showed that the R9 peptide formed stable complexes with plasmid and retarded the pDNA movement in gel electrophoresis at N/P ratios equal to and higher than 15. FESEM microscopy findings demonstrated that the morphology of these R9-pDNA complexes was nearly spherical and was in the N/P ratio of 30 and 45. The size of these particles reduced approximately from 80 nm at N/P ratio of 30 to 40 nm at N/P ratio of 40. shRNA suppressed the E6 gene expression by approximately 28.76% when transfected with R9 compared to 52.39% when transfected with PEI. This reduction led to a significant targeted toxicity on HeLa cells, indicating a potential therapeutic application for these complexes.

Togtema et al.^[^^20^^]^ reported that E6-specific siRNA inhibition of E6 expression in HPV16-infected cells in the presence of active p53 and active E7 led to apoptosis by causing irregular cell proliferation. Furthermore, several studies have suggested that p53 and Rb tumor suppressor pathways are intact in HeLa cells, and activating these pathways through the suppression of HPV E6 and E7 can lead to the delivery of growth inhibitory signals to the cells^[^^20^^-^^23^^]^. Butz et al.^[^^23^^]^ demonstrated that both vector-borne and synthetic siRNAs, directed against the HPV E6 oncoprotein of HeLa cells and in the presence of E7 oncoprotein, restored the activity of tumor suppressor pathways and resulted in apoptotic cell death. Chang et al.^[^^24^^]^ suggested that the inhibition of E6 by siRNA causes a 37% decrease in gene expression in HeLa cells compared to the control group. In another study, Wang and colleagues utilized irreversible electroporation to transfer shRNA plasmids to knockdown E6 gene of HPV18 in HeLa cells, with up to 90% knockdown efficiency in vitro. They demonstrated a significant inhibition of tumor growth in vivo^[^^17^^]. ^This report highlights the important effect of the delivery method on the final outcome of oncogene suppression by shRNA. The transfection efficiency also influenced the effectiveness of shRNA as more efficient transfection of HeLa cells by PEI resulted in more profound suppression of E6 expression compared to R9. However, this polymer exhibited a great toxic effect on cells, which is undesirable^[^^25^^]^.

Viral vectors have been utilized to deliver interference RNAs to mammalian cells. Anastasov et al.^[^^26^^] ^used lentiviral vectors for shRNA delivery to B and T lymphoma cells, with a suppression rate of 80%. However, despite their efficiency, lentiviral vectors, the same as gene delivery agents, have some disadvantages, such as insertional mutagenesis and immune response. This demerits enforced the research on non-viral vectors, including CPPs. Previous studies have explored the potential of arginine-rich CPPs for delivery of plasmids to mammalian cells. Investigators have reported a stable nano-complex formation at the N/P ratio higher than 15^[^^16^^]^, which was also observed in our study. Ideally, CPP-siRNA nanocomplexes should be less than 200 nm in size to achieve a proper diffusion into tumor tissue and have an optimal endocytic uptake via the enhanced permeation and retention effect^[^^15^^]^. In the present study, the mean diameter of the nanocomplexes were 80 nm at N/P ratios of 30. Furthermore, based on the FESEM images, the structure of the prepared nanocomplexes became smaller and more uniform when the N/P ratio increased from 15 to 45. However, the morphological transition from N/P of 30 to 45 did not affect the transfection efficiency of the nano-complexes. Alhakamy et al.^[^^27^^,^^28^^]^ reported that R9 transfection efficiency was not as well as that of the widely used PEI transfection reagent, which is in accordance with our observation. To enhance the transfection efficiency of these peptides, the mentioned authors evaluated the use of CaCl_2_ in their formulation, which lead to a decrease in the particle size from 800 nm to about 300 nm and an increase in the transfection efficiency in 293T cells. However, the added salt might likely be diluted in body fluids and cannot exert the same effect in vivo*.* Chemical modification of R9 peptide has also been reported where cholesterol and cysteine were conjugated to this peptide to enhance its transfection efficiency^[^^29^^,^^30^^]^. These modifications will probably change other biological properties of R9, which needs to be further studied. Herein, despite the lower transfection efficiency of R9, which was reflected in lower luciferase expression and lower E6 oncogene suppression, the final desired outcome of the targeted cell toxicity was not significantly lower than PEI. This observation suggests no direct relationship between E6 suppression and cell toxicity^[^^20^^,^^31^^]^. Therefore, it could be concluded that the various downstream pathways associated with P53 deactivation may influence the final observed toxicity. Co-suppression of those pathways via multiple shRNA vectors can have a synergistic effect on cell toxicity. 

In summary, the R9 peptide could efficiently condense, stabilize and transfect E6 shRNA in vitro. E6 shRNA transfected with R9 suppressed E6 expression with at least half the efficiency of PEI. The lower E6 suppression level, did not lead to lower targeted toxicity compared to PEI. Together, our findings suggest that R9 is appropriate for being applied as a non-viral gene carrier. Further investigations are necessary to explore the use of multiple shRNA sequences and design, as well as develop other Tat-derived arginine-rich peptides to optimize the delivery efficiency of these vectors.

## DECLARATIONS

### Ethical statement

Not applicable.

### Data availability

The raw data supporting the conclusions of this article are available from the corresponding author upon reasonable request.

### Author contributions

RT: investigation, visualization, writing original draft, and preparation; EA: data curation; AA: formal analysis; NM: conceptualization, funding acquisition, supervision, Writing, review and editing. All the authors have read and approved the final version of the manuscript.

### Conflict of interest

None declared.

### Funding/support

This work was funded by the Pasteur Institute of Iran (grant number: 934 and Ph.D. thesis grant number: BP-9475) and the Iran National Science Foundation (INSF) (grant number: 97011564).
